# Seroevidence of SARS-CoV-2 spillback to rodents in Sarawak, Malaysian Borneo

**DOI:** 10.1186/s12917-024-03892-5

**Published:** 2024-04-27

**Authors:** Cheng Siang Tan, Madinah Adrus, Sultana Parvin Habeebur Rahman, Haziq Izzuddin Muhamad Azman, Riz Anasthasia Alta Abang

**Affiliations:** 1 Faculty of Medicine and Health Sciences, Universiti Malaysia Sarawak, Kota Samarahan, Sarawak, 94300 Malaysia; 2 Faculty of Resource Science and Technology, Universiti Malaysia Sarawak, Kota Samarahan, Sarawak, 94300 Malaysia

**Keywords:** SARS-CoV-2, Rodents, Spillback, Reverse zoonosis, Neutralising antibodies

## Abstract

**Background:**

SARS-CoV-2 is believed to have originated from a spillover event, where the virus jumped from bats to humans, leading to an epidemic that quickly escalated into a pandemic by early 2020. Despite the implementation of various public health measures, such as lockdowns and widespread vaccination efforts, the virus continues to spread. This is primarily attributed to the rapid emergence of immune escape variants and the inadequacy of protection against reinfection. Spillback events were reported early in animals with frequent contact with humans, especially companion, captive, and farmed animals. Unfortunately, surveillance of spillback events is generally lacking in Malaysia. Therefore, this study aims to address this gap by investigating the presence of SARS-CoV-2 neutralising antibodies in wild rodents in Sarawak, Malaysia.

**Results:**

We analysed 208 archived plasma from rodents collected between from 2018 to 2022 to detect neutralising antibodies against SARS-CoV-2 using a surrogate virus neutralisation test, and discovered two seropositive rodents (*Sundamys muelleri* and *Rattus rattus*), which were sampled in 2021 and 2022, respectively.

**Conclusion:**

Our findings suggest that *Sundamys muelleri* and *Rattus rattus* may be susceptible to natural SARS-CoV-2 infections. However, there is currently no evidence supporting sustainable rodent-to-rodent transmission.

## Main

The severe acute respiratory syndrome coronavirus 2 (SARS-CoV-2), the virus that causes the coronavirus disease 2019 (COVID-19) was first reported in a cluster of patients with pneumonia in Wuhan, China, in late 2019. SARS-CoV-2 spreads rapidly, resulting in clusters of respiratory infections worldwide, and been declared a pandemic by the World Health Organisation (WHO) in March 2020. As of 19th September 2022, SARS-CoV-2 infected > 617 million individuals and claimed > 6.5 million lives worldwide [1].

The SARS-CoV-2 spillover event is considered one of the greatest One Health disasters that costed >$16 trillion USD in pandemic management, reduced productivity and health [2]. Retrospectively, several epidemiological warnings over the course of the last two decades in the form of epidemic coronavirus spillover events, namely the Severe Acute Respiratory Syndrome Coronavirus (SARS-CoV) in 2002 and Middle Eastern Respiratory Syndrome Coronavirus (MERS-CoV) in 2012 were significantly downplayed. Following this, the discovery of a bat coronavirus RaTG13 with a 96.2% genomic sequence similarity to SARS-CoV-2 in Mojiang, Yunnan, China in 2012 failed to ring a bell [3]. Recently, another bat coronavirus, BANAL-13, with higher genomic similarity to SARS-CoV-2 (96.8%), has been discovered in Laos, further cementing the idea that SARS-CoV-2 was indeed a spillover from bats. To date, the intermediary host of SARS-CoV-2 remains a mystery, although several studies have suggested pangolins to be the probable intermediate host [4–6].

The first documented spillback case was recorded in Hong Kong on the 29th February 2020 involving a dog [7]. Since then, SARS-CoV-2 has been reported in twenty-three different animal species globally, including cats, dogs [8], mink, otters, pet ferrets, lions, tigers [9], pumas, snow leopards, gorillas, white-tailed deer [10, 11], fishing cat, Binturong, South American coati, spotted hyena, Eurasian lynx, Canada lynx, hippopotamus, hamster, mule deer, giant anteater, West Indian manatee, black-tailed marmoset, common squirrel monkey [12] (as of 31st July 2022).

Historically, scientific evidence has shown that rodents are non-competent hosts for SARS-CoV-2 primarily due to the low avidity of the wildtype SARS-CoV-2 receptor binding domain (RBD) to murine Angiotensin-Converting Enzyme 2 (ACE2) expressed in cell lines [12–14]. However, with the emergence of the N501Y mutation in the spike of Alpha/B.1.1.7 (WHO/Pango lineage), Beta/B1.351 and Gamma/P3 variants of concern, there has been a notable shift. This mutation not only confers enhanced affinity towards human ACE2 but also to house mice (*Mus musculus*) and brown rats (*Rattus norvegicus*) [15]. To date, there has been no active SARS-CoV-2 infections in rodents. The sole evidence of SARS-CoV-2 exposure in rodents comes from Hong Kong, where seropositivity was identified in a single individual (*Rattus norvegicus*) [16]. Similar serosurveillance studies conducted in Belgium and Germany have reported no prior exposure of rodents to SARS-CoV-2 [17, 18].

With the increasing trend of home quarantine for SARS-CoV-2 infected individuals, thereby replacing designated quarantine centres, and the high prevalence of subclinical infections, we hypothesize that more infectious SARS-CoV-2-contaminated waste may enter unsegregated domestic waste. This situation could potentially expose scavenging rodents to SARS-CoV-2 infections. Therefore, our study aims to investigate the presence of SARS-CoV-2 neutralising antibodies in wild rodents in Sarawak, Malaysia.

## Results

**Fig. 1:**
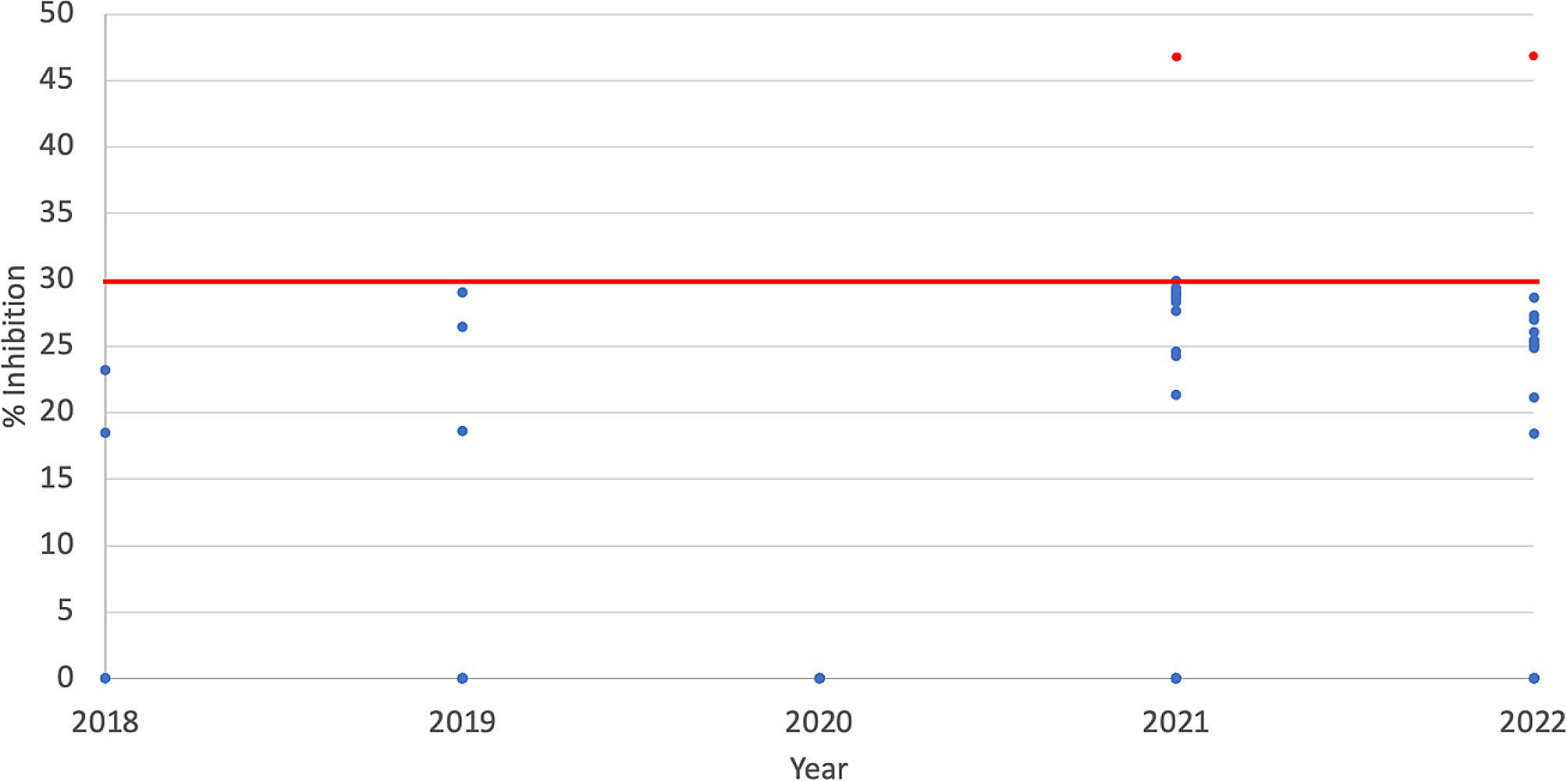
Anti-SARS-CoV-2 neutralising antibody in rodents sampled from 2018 to 2022. Neutralising antibody level in % inhibition was derived from the surrogate virus neutralisation test cPASS (Genscript) and the manufacturer’s positive cutoff value was represented as a red line. Blue and red dots represent seronegative and seropositive results, respectively

A total of 208 archived plasma samples from rodents, collected between 2018 to Aug 2022 in Sarawak, Malaysia, underwent screening for neutralising antibodies against SARS-CoV-2. The tested plasma samples included rodents from six species: *Rattus tanezumi*, *Sundamys muelleri, Rattus tiomanicus, Rattus rattus, Leopoldamys sabanus*, and *Maxomys whiteheadi* (Table 1). Notably, one individual each from the year 2021 (*Sundamys muelleri*) and year 2022 (*Rattus rattus*) exhibited surrogate virus neutralisation test (sVNT) inhibition of 47% surpassing the manufacturer’s positive cutoff value ≥30% (see Fig. 1). The seropositivity of SARS-CoV-2 in the rodent was 0% (*n* = 0/4; 2018), 0% (*n* = 0/55; 2019), 0% (*n* = 0/40; 2020), 0.01% (*n* = 1/85; 2021), *n* = 0.04% (1/24; 2022).

## Discussion

### Significance of findings

We provided the first serological evidence of SARS-CoV-2 exposure in *Sundamys muelleri* and *Rattus rattus.* In a related study, Miot et al., 2022 reported the detection of SARS-CoV-2 neutralising antibodies in *Rattus norvegicus*, suggesting potential susceptibility of SARS-CoV-2 within the Muridae family [19]. Notably, these three species – *Sundamys muelleri, Rattus rattus* and *Rattus norvegicus* - are free-ranging ubiquitous wildlife (synanthrope) that have adapted to urban living. They typically manifest as pests, displaying primarily nocturnal behaviour and shying away from humans, unlike many other naturally infected animal species by SARS-CoV-2.

### Gradual adaptation SARS-CoV-2 RBD to murine ACE2

The initial study indicated that the prototype SARS-CoV-2 RBD exhibits poor binding to murine ACE2 suggesting a natural resistance of rodents to SARS-CoV-2 infections [14]. However, subsequent in silico and in vivo studies have demonstrated the gradual adaptation of SARS-CoV-2 variants to murine ACE2 receptors. For instance, genomic sequence analysis suggests that the SARS-CoV-2 Omicron variant may have been resulted from a ping pong effect – a spillback from humans to mice, garnered mutation that enhanced the RBD’s avidity for the mouse ACE2 receptor before spillover again into humans [20, 21]. In vivo studies have demonstrated the permissiveness of deer mice (*Peromyscus manicalatus*) and golden/Syrian hamster (*Mesocricetus auratus*) to SARS-CoV-2 infections in laboratory settings, after being inoculated with infective doses ranging from 10^4^ to 10^6^ tissue culture infective dose 50 (TCID_50_) [22, 23], a high inoculum titre that may not be naturally encountered in the habitat. Infected deer mice shed viruses in the respiratory tract, faeces and urine; and they readily transmit the infection to naïve deer mice via direct contact [23] highlighting a possible risk of sustained mouse-to-mouse transmission.

The observed low SARS-CoV-2 seroprevalence in our study suggests that spillback from humans to rodents via environmental contamination does occur but is not sustainable within the free-ranging rodents population. Permissive hosts such as white-tailed-deer [10], minks [24] and Syrian hamster [25] exhibited high seroprevalence rates with high sVNT inhibition, indicating ongoing active transmission at the time of sampling. The moderate sVNT inhibition observed in our study may represent the waxing or waning of immunity after exposure.

### Persistence of SARS-CoV-2 in the environment

Infected individuals shed the SARS-CoV-2 virus via their respiratory tract and faeces. Since the primary mode of transmission is via aerosol, the use of face masks and respirators have been proven effective in reducing the transmission of SARS-CoV-2 and other respiratory pathogens. Face masks absorb infectious respiratory droplets, effectively reducing the release of infectious aerosols into the surrounding but in one fallen swoop enriches it with infectious particles [26]. Since SARS-CoV-2 can survive on inanimate objects for hours, and even days [27], and this survival period can be extended as long as 21 days in the presence of protective biological fluids such as nasal mucus, sputum and saliva [28]. Consequently, the improper disposal of used facemasks has become a concern, but has become largely neglected practice in public health settings [29]. In addition, the increased usage of rapid SARS-CoV-2 Antigen Test (RAT) kits has contributed to the rise in the disposal of used RAT kits in unsegregated domestic waste. Rodents scavenging through domestic waste may inadvertantly come into contact with used facemasks and RAT kits, which could potentially serve as a source of transmission.

Both SARS-CoV-1 and SARS-CoV-2 are known to be shed in the stool of some infected individuals, raising concerns about potential over faecal-oral transmission. Although viable SARS-CoV-2 has been isolated from the stool [28], there has been no documented evidence of faecal transmission. Despite these arguments, SARS-CoV-2 remains viable in the faeces, contaminating sewer shed and leading to the discovery of both circulating [28, 30] and novel SARS-CoV-2 lineages in the watershed [28]. Smyth and co-workers have argued that the mysterious SARS-CoV-2 lineages exhibit rare mutations which allow expanded tropism to cells expressing human, mouse or rat ACE2 receptors, suggesting a potential origin of these novel SARS-CoV-2 lineages from rodents [31].

The two seropositive rodents were sampled in market and residential areas during the Delta and Omicron variants wave, respectively [32, 33]. Both locations in the context of One Health could be the nexus of the spillback events, with used face masks, RAT kits, and other potentially infectious materials entering the domestic refuse bins until scheduled disposal. We speculate that the infected rodents may have scavenged the SARS-CoV-2 contaminated food waste in the exposed municipal bins due to spoilage, negligence or overflowing refuse.

### Limitations

While we believe that this manuscript describes the first detection of neutralising SARS-CoV-2 antibodies in rodents in Malaysia, we also recognised several limitations in our study [1]. Neither virological nor molecular screenings were carried out in an attempt to confirm the infecting agent, which does not fulfil Koch’s postulates [2]. The moderate sVNT inhibition could indicate potential waxing or waning of immunity, non-specific antibody binding, or cross-reactivity [10]. However, plasma obtained from the pre-COVID-19 era (2018–2019), and low COVID-19 transmission era (2020) discounted the possibility of non-specific binding and cross-reactivity, [3] We did not compare our findings with the ‘gold standard’ PRNT50 due to the absence of biocontainment facilities. Nevertheless, the species-independent sVNT has consistently shown high concordance with VNT or PRNT50. However, previous assay evaluation studies have reported that the sensitivity of sVNT may differ between species. For instance, higher sVNT inhibition was observed on low PRNT50 sera from ferrets, rabbits, and cattle, while negative to moderate PRNT50 sera from a non-human primate, *Cynomolgus macaques* yielded low to negative sVNT inhibition [34]. Nonetheless, sVNT has been consistently shown to be in concordance with PRNT50 in humans, dogs, cats and hamsters [4, 35, 36] archived plasmas were collected opportunistically and may have introduced sampling bias.

## Conclusions

The spillback of SARS-CoV-2 to rodents has been happening in Sarawak, Malaysian Borneo since 2021, but there is no evidence of a sustained transmission within the rodents’ population.

Continuous surveillance should be intensified at strategic locations such as municipal landfill and sewerage treatment plant to ensure that public health mitigation procedures can be implemented in a timely manner if a sustained transmission is detected.

## Methods

### Samples

Archived plasma samples from rodents collected for an ongoing pathogen surveillance activity from Jan 2018 to August 2022 were used in this study [37]. Cage traps were deployed at research sites during the sampling periods. The traps were baited with banana, salted fish, shrimp paste, and bread. Regular checks of the cage traps were done twice daily, both in the morning and evening, with bait replenishment carried out each morning. Rodents captured in the traps were carefully secured in cloth bags and subsequently transported to the laboratory for additional processing.

The morphometric measurements of each captured rodents were recorded, such as the total length, head-body length, tail length, and hindfoot, together with their weight in the laboratory. The species identification was done in accordance with the criteria outlined in Phillipps and Phillipps, 2018 [38].

The rodents were anesthetized in a closed container permeated with excess isoflurane. Subsequently, once the animals were confirmed deceased, blood samples were extracted from the heart using a 5 ml syringe via the intracardiac route, and stored in EDTA tubes. Blood and plasma were separated via centrifugation, and stored at -20 °C until use.

**Table 1:** Rodents species captured in forested, residential, and market areas in Sarawak

	Total number of rodent individuals capture (number of seropositive individual for SARS-CoV-2)																					
	Forested Areas^a^		Residential Areas^b^								Market Areas^c^											
Species name (Common name)	**GGNP**	**KNP**	**KM**	**KB**	**SJ**	**PJ**	**UNS**	**TMR**	**RHP**	**B4**	**SMR**	**KBR**	**WFK**	**PSM**	**BKS**	**BTW**	**PTK**	**PPM**	**PST**	**FRM**	**PNM**	**Total**
*Leopoldamys sabanus* (Long-tailed giant rat)	1	1	-	-	-	-	-	-	-	-	-	-	-	-	-	-	-	-	-	-	-	2
*Maxomys whiteheadi* (Whitehead’s spiny rat)	-	1	-	-	-	-	-	-	-	-	-	-	-	-	-	-	-	-	-	-	-	1
*Rattus rattus* (House rat)	-	-	-	-	-	-	-	-	-	3 (1)	-	-	-	-	-	-	-	-	-	-	-	3
*Rattus tanezumi* (Asian house rat)	1	-	1	3	7	6	18	9	13	2	1	5	15	14	2	6	5	-	9	10	17	144
*Rattus tiomanicus* (Malaysian field rat)	-	-	-	-	18	-	1	1	1	-	-	-	-	-	1	-	-	-	-	2	-	24
*Sundamys muelleri* (Muller’s rat)	-	1	-	-	-	19	6	2	-	-	-	-	-	1	-	2	-	2 (1)	-	1	-	34
Total	2	3	1	3	25	25	25	12	14	5	1	5	15	15	3	8	5	2	9	13	17	208

^a^Two forested areas in Sarawak (GGNP, Gunung Gading National Park and KNP, Kubah National Park)

^b^Eight residential areas in Sarawak (KM, Meranek Village, Kota Samarahan; KB, Baru Village, Kota Samarahan; SJ, Sadong Jaya; PJ, Petrajaya, Kuching; UNS, Universiti Malaysia Sarawak; TMR, Taman Riveria, Kota Samarahan; RHP, RH Plaza, Kuching; B4, 4th Mile, Kuching)

^c^Eleven market areas in Sarawak (SMR, Semarak Market, Kuching; KBR, Kubah Ria Market, Kuching; WFK, Waterfront Kuching; PSM, Semariang Market, Kuching; BKS, Bandar Baru Riyal Market, Kota Samarahan; BTW, Bintawa Market, Kuching; PTK, Kenyalang Park Market, Kuching; PPM, Pusat Penjaja MPKS, Kota Samarahan; PST, Stutong Market, Kuching; FRM, Farley Market, Kuching; and PNM, Petanak Market, Kuching)

### Surrogate virus neutralisation test

All samples were screened for the presence of neutralising antibodies targeting the SARS-CoV-2 Wuhan-Hu-1 (ancestral) RBD using species-independent surrogate virus neutralisation test, cPASS™ (Genscript, NJ, USA) [39]. Briefly, the tenfold diluted plasmas were combined with equal volume of horseradish peroxydase (HRP) conjugated RBD and preincubated for 30 min at 37 °C, before being transferred to ACE2-coated ELISA plate and incubated for 15 min at 37 °C. The wells were sequentially washed four times and chromogenic signal development was done using tetramethylbenzidine (TMB) substrate and reaction stopped with concentrated 1 M hydrochloric acid. Absorbance was measured at 450 nm using SpectraMax iD3 microplate reader (Molecular Devices, San Jose, USA). Results are presented as percent inhibition = (1- [OD value of Sample/OD value of Negative control]) x 100%. The manufacturer’s positive cutoff value is ≥ 30%. Results were analysed in Microsoft Excel Version 16.62.

## Data Availability

Data used and materials used in this manuscript can be obtained from the corresponding author upon reasonable request.

## Abbreviations

ACE: angiotensin converting enzyme
CoV: coronavirus
COVID: coronavirus disease
ELISA: enzyme-linked immunosorbent assay
HRP: horse radish peroxidase
MERS: middle eastern respiratory syndrome
PRNT: plaque reduction neutralisation test
RAT: rapid antigen test
RBD: receptor binding domain
SARS: severe acute respiratory syndrome
sVNT: surrogate virus neutralisation test
TCID: tissue culture infective dose
TMD: tetramethylbenzidine
USD: united states dollar
VNT: virus neutralisation test
WHO: World Health Organisation
